# Availability of Donor Milk for Very Preterm Infants Decreased the Risk of Necrotizing Enterocolitis without Adversely Impacting Growth or Rates of Breastfeeding

**DOI:** 10.3390/nu11081895

**Published:** 2019-08-14

**Authors:** Débora Cañizo Vázquez, Sandra Salas García, Montserrat Izquierdo Renau, Isabel Iglesias-Platas

**Affiliations:** 1 Neonatology Department, Hospital Sant Joan de Déu, Universidad de Barcelona, BCNatal, 08950 Esplugues de Llobregat, Barcelona, Spain; 2 Neonatology Department, Hospital General Universitari Castelló, 12004 Castelló de la Plana, Spain

**Keywords:** preterm infant, human milk, donor human milk, formula feeding, breastfeeding, necrotizing enterocolitis, growth

## Abstract

Human milk contains non-nutritional factors that promote intestinal maturation and protect against infectious and inflammatory conditions. In the Neonatal Intensive Care Unit (NICU) setting, donor milk (DM) is recommended when availability of own mother’s milk (OMM) is not enough. Our aim was to compare the incidence of necrotizing enterocolitis (NEC) and late-onset sepsis (LOS) in very preterm infants (VPI) after the introduction of DM. Growth and breastfeeding rates were examined as secondary outcomes. Single center, observational and retrospective cohort study comparing 227 VPI admitted to our neonatal unit before (Group 1, *n* = 99) and after (Group 2, *n* = 128) DM introduction. Enteral nutrition was started earlier after DM availability (2.6 ± 1.1 vs. 2.1 ± 1 days, *p* = 0.001). Incidence of NEC decreased in group 2 (9.1% vs. 3.4%, *p* = 0.055), especially in those born between 28 and 32 weeks (5.4 vs. 0.0%, *p* = 0.044). Surgical NEC was also less frequent. Suffering NEC was 4 times more likely in group 1 (multivariate analysis). Availability of DM did not impact breastfeeding rates or preterm growth. Our findings support the protective role of DM against NEC, particularly in non-extreme VPI, a group less frequently included in clinical guidelines and research studies on the use of DM.

## 1. Introduction

The benefits of breastmilk for both mother and infant are well established [1,2]. Breast milk should be the first choice for feeding premature and low-birth weight newborns. Necrotizing enterocolitis (NEC) and late-onset sepsis (LOS) are infectious- inflammatory diseases of premature infants with a high rate of mortality, even today [3,4]. Breast milk has been shown to act as a preventative factor [5,6] and it is postulated that this is through several non-nutritional factors, such as immunoglobulins, growth factors and substances with antioxidant capacity [7,8]. Some of these compounds play a role in modulation of the immune system and in the pathophysiology of these and other diseases [7,8,9,10,11].

Establishing and maintaining an appropriate milk supply after preterm birth comes with its own challenges, including maternal illness, the need for artificial expression and stress surrounding separation from and worry about the well-being of the child [12]. International scientific societies recommend donor milk (DM) as the first alternative when the available quantity of own mother’s milk (OMM) is not enough to cover the nutritional requirements of the premature infant [13]. It is unclear whether the advantages of pasteurized human milk can be similar to those of OMM. Pasteurization does not alter caloric or macronutrient content, but there is controversy about how it does affect other biologically active components, such as IgA, lysozyme, lactoferrin, lymphocytes, lipase, alkaline phosphatase, cytokines (like IL10), growth factors and antioxidant capacity [8,14,15,16]. Several studies have shown a protective effect of DM against NEC [6,17] and an improvement in feeding tolerance [7,18] when comparing with formula feeding, and systematic reviews of published data find a decreased incidence of bronchopulmonary dysplasia (BPD) [19] and LOS [6]. Some even suggest better neurodevelopmental and cardiovascular outcomes with the use of DM [20,21].

Although growth of premature infants fed DM (especially if unfortified) might be slower when compared to formula-fed counterparts, no long-term nutritional compromise has been described [7]. Another concern that arose with the use of milk banks was that donor milk could threaten the motivation of the staff to provide support or the commitment of mothers to provide milk for their infants, but this does not seem to be the case [22].

The aim of this study was to compare the incidence of clinical complications (NEC and LOS) in very preterm infants (VPI) (≤32 weeks gestational age at birth) before and after the introduction of DM instead of artificial formula to supplement OMM when necessary. Rates of growth and breastfeeding in both groups were examined as secondary outcomes.

## 2. Materials and Methods

### 2.1. Study Design

Single center, observational retrospective cohort study of VPI admitted to a level III intensive care unit. The cohorts were defined by the use of premature artificial formula (Group 1) or donor milk (Group 2) for enteral feeding in the absence of enough OMM. Sample size was calculated to detect a reduction in the incidence of a composite outcome of NEC or LOS to a third (based on literature reports) of the basal figure of about 25% in our population of VPI. The estimated sample size was 82 patients per group for a confidence level of 95% with an 80% statistical power.

All subjects gave their informed consent for inclusion before they participated in the study. The study was conducted in accordance with the Declaration of Helsinki, and the protocol was approved by the local Ethics Committee (Fundació Sant Joan de Déu Ethics Committee; PIC-20-16).

### 2.2. Patients

Babies born at or before 32 completed weeks of gestational age were considered eligible. Inclusion criteria: Admission before 24 h of life and survival for longer than a week. Exclusion criteria: Major congenital malformations, chromosomal, genetic or metabolic abnormalities or the absence of clinical records. Group 1 comprised the 2 years before (2009–2010) and group 2 the 2 years after (2012–2013) the introduction of DM in our unit. The year of overlap (2011) was considered as an implementation period and not taken into consideration for the analysis. Clinical and growth variables as well as nutritional supplies were extracted from clinical charts.

### 2.3. Clinical Protocols

Nutritional management of VPI in our unit follows local written guidelines in accordance to international recommendations. In short and as previously described [23], parenteral nutrition (PN) is started immediately after birth through a central line for the provision of 2.5 g/kg of protein and 62 kcal/kg, with stepwise increases reaching 3.5–4 g/kg of protein and 100 kcal/kg depending on metabolic tolerance and progression of enteral feeding. Enteral nutrition is started as soon as possible depending on clinical condition of the patient. Own mother’s milk is the first option. The difference between groups 1 and 2 was the supplementation with a preterm formula (Alprem^®^, Nestlé, Switzerland) or DM respectively, when the volume of enteral feeding prescribed was higher than the mother´s milk supply. Donor milk is maintained if needed until one month of age if the baby is born under 28 weeks or 1000 g and during the first 3–7 days in newborns 28–32 weeks that are over 1 kg. All human milk was fortified (Enfamil^®^ Human Milk Fortifier Powder, Mead Johnson, Chicago, IL, USA) from an intake of 80–100 mL/kg/day.

There were no other changes in clinical protocols for any other areas of care in the unit during the study period.

### 2.4. Study Variables

Nutrition and growth: Volumes of enteral and parenteral nutrition administered were extracted from clinical charts. Macronutrients were calculated assuming standard compositions of preterm and term milk [24] or from manufacturer´s information and considering the PN prescription. Nutritional supply was recorded daily for the first 14 days of life and again at 28 days and at 36 weeks postmenstrual age (PMA). Information on start of enteral feeding, achievement of full enteral nutrition and rates of OMM at 7, 14, 28 days of life, at full enteral nutrition and at discharge was also registered.

Weight was evaluated at admission, day 14 and day 28 of life and at 36 weeks PMA. We also collected data on minimum weight, days to maximum initial weight loss and days to regain birth weight. Length and head circumference (HC) data were available at birth and discharge. To allow for comparisons at different gestational ages, measurements of anthropometric parameters were transformed into z-scores for gestational age using local intrauterine growth standards [25,26]. 

Clinical variables: The main complications of prematurity were studied as clinical outcomes. A baby was considered to have NEC if fulfilling criteria compatible with Bell´s stage 2 or higher [27]. Late-onset sepsis was defined as the presence of a positive culture of blood or a sterile fluid on or after the fourth day of life. Intraventricular hemorrhage was graded according to Papile et cols [28]. Patent ductus arteriosus (PDA) was diagnosed as the presence of clinical signs (heart murmur, hyperdynamic precordial impulse, full pulses, widened pulse pressure, and/or worsening of the respiratory status) with a ductal right-to-left shunt in the echocardiography. Bronchopulmonary dysplasia was defined as need for oxygen for more than 28 days. Retinopathy of prematurity (ROP) was staged according to the International Committee for classification of ROP [29] and considered as severe if requiring laser therapy. 

### 2.5. Statistical Analysis

All data were analyzed with the SPSS^®^ (Statistical Package for Social Sciences, IBM, Chicago, IL, USA) software, v17. Qualitative variables were expressed as frequencies or percentages and quantitative variables as means and standard deviations. Comparisons between Groups 1 and 2 were performed by chi-square and student *t* tests as appropriate. Differences were considered significant if *p*-values were <0.05. Logistic regression models were used to analyze the risk of NEC in Groups 1 and 2 while adjusting for relevant covariates.

A secondary analysis for the groups ≤28 weeks and >28 weeks was performed on the basis of a higher risk of developing NEC among the extremely premature babies and because, as stated before, the protocol for administration of donor milk in our unit was different in the under and over 28 weeks groups (longer duration in the former).

## 3. Results

### 3.1. Description of the Sample

**Figure 1:**
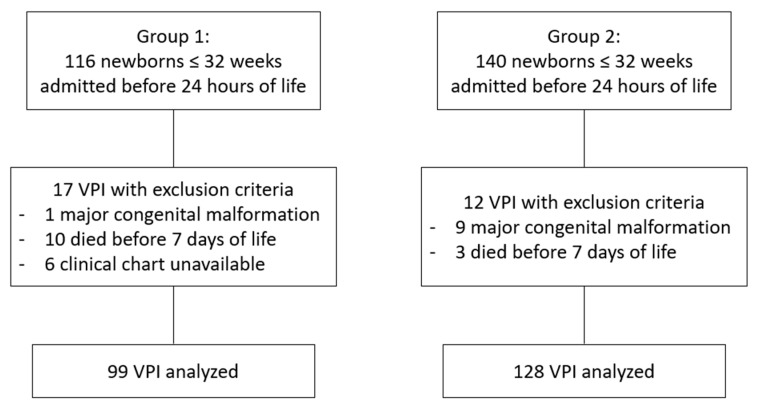
Flowchart of study participation.

A total of 256 VPI were admitted to our unit during their first 24 h of life in the study period. Of them, 23 had exclusion criteria, leaving a total of 227 for analysis, 99 in Group 1 (before availability of DM) and 128 in Group 2 (after availability of DM) (Figure 1). Basal characteristics of the sample are summarized in Table 1. There were no differences between groups regarding gestational age, gender, multiparity, cesarean section, percentage of children with intrauterine growth restriction or severity of illness on admission as assessed by CRIB (Clinical Ric Index for Babies) score. Birth weight was slightly lower in Group 2, with no differences in z score (Table 1).

### 3.2. Enteral Nutrition Was Started Earlier after Availability of DM

**Figure 2:**
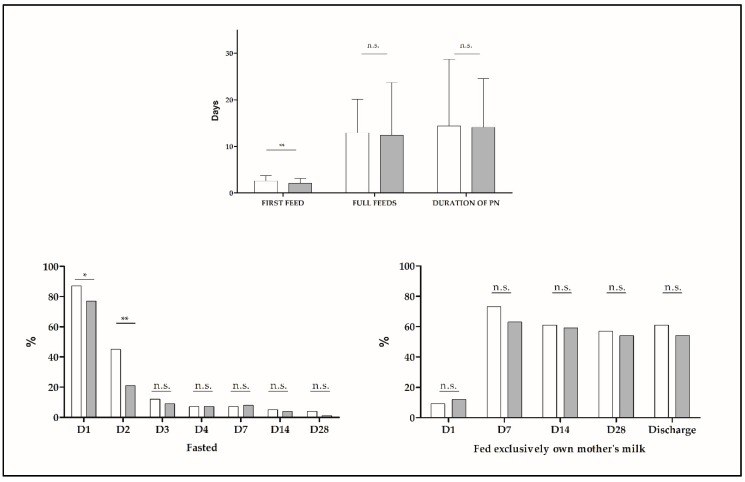
Summary of nutritional characteristics of very preterm infants (VPI) in G1 (white) and G2 (grey). * *p *< 0.05; ** *p *< 0.001; n.s.: non-significant (*p*-value > 0.05). G1: Group 1, G2: Group 2, D: Day of life, PN: Parenteral nutrition.

The start of enteral nutrition happened about half a day earlier in Group 2 (2.6 ± 1.1 vs. 2.1 ± 1.0 days, *p* = 0.001) and the percentage of fasted babies was lower on days of life 1 and 2 in Group 2. This did not impact age or milk volume at full enteral nutrition, days on parenteral nutrition or milk volumes fed during the 1st and 2nd weeks of life or at day 28 (Figure 2). There was a trend for a higher percentage of patients receiving only human milk on days 14 and 28 of life after the introduction of DM (65.2% vs. 76.9%, *p* = 0.066 and 62.9% vs. 75.2%, *p* = 0.065 respectively). This was significant for babies ≤ 28 weeks on day 14 (75.0% vs. 100.0%, *p* = 0.004). Nutrition during the second week of life and thereafter was homogenous between groups. During the first week we found small but significant differences in parenteral nutrition. Total fluid volume was higher in Group 1 (104.6 ± 11.0 vs. 96.3 ± 15.2, *p* < 0.001) and intravenous lipids (1.6 ± 0.7 vs. 1.9 ± 0.6, *p* = 0.014) were higher in Group 2. Total calories (78.4 ± 8.8 vs. 81.0 ± 9.1, *p* = 0.035) and protein (2.9 ± 0.4 vs. 3.0 ± 0.4, *p* = 0.096) were higher in Group 2.

### 3.3. Breastfeeding Rates Did Not Change with the Introduction of Donor Milk 

The percentage of children receiving their OMM was the same in both groups (Table 2). We have a rate of exclusive breastfeeding at discharge of 56.8% (60.6% in ≤28 weeks and 53.9% in >28 weeks).

### 3.4. Rates of Early Growth Were Better in Group 2

The percentage of weight loss immediately after birth was smaller in Group 2, due to differences in patients ≤28 weeks at birth (Table 2). There were no differences in age at minimum weight or days to recover birthweight. Fall in weight z-scores at 28 day of life (dol) was smaller in Group 2 than in Group 1, while no changes were seen at 36 weeks of PMA or discharge. In a multivariate analysis by linear regression, the group in which the patient was born was one of the determinants for fall in weight z-score at 28 dol after adjustment for confounders (Table S1). There were no differences between sexes in the growth and nutritional outcomes analyzed (data not shown).

### 3.5. The Incidence of Necrotizing Enterocolitis Decreased after the Introduction of DM

We found no differences between groups regarding ventilatory support, incidence of BPD, severe retinopathy or intraventricular hemorrhage. The rate of LOS was similar between groups and the same was true for duration of antibiotic treatment, days of central line and parenteral nutrition (Table 3). The incidence of NEC was slightly lower in Group 2 (9.1% vs. 3.4%, *p* = 0.055), especially in the group with a gestational age between 28 and 32 weeks at birth (5.4 vs. 0.0%, *p* = 0.044). Mortality was similar in both groups (4.0% vs. 5.5%, *p* = 0.619), but a history of NEC tended to be more frequent among very preterm babies that died in Group 1 (75.0% vs. 14.3%, *p* = 0.088). Surgical NEC was more frequent in G1 (5/99, 5.0% vs. 1/128, 0.8%), but this was not statistically significant (*p* = 0.308). An analysis of the risk of NEC in both groups including possible confounders showed that the odds of suffering NEC was 4 times higher before the introduction of DM (Table S2). There were no differences between boys and girls (data not shown).

## 4. Discussion

Enteral feeding with DM when own mother’s milk is not available or is not enough has been associated with reduced mortality and a decrease in morbidity of VPI [6,18,19]. However, there is a concern about nutritional requirements of VPI fed with DM because artificial formula results in higher rates of weight gain and linear growth due to its greater amounts of nutrients. Preterm formulas are energy-enriched and variably protein and mineral-enriched when compared to mature human milk, and the nutrient content of donor milk may be further compromised by pasteurization [30]. Nevertheless, the role of an exclusive human milk diet is well recognized in the prevention of NEC and other severe complications like invasive infections. In a recent publication, this effect has been related to the presence of antioxidants in breast milk, and these could be impaired by DM processing [11].

Nowadays it remains unclear if DM has the same advantages as OMM and may have some disadvantages related to growth with respect to artificial formula. Our aim was to compare short-term outcomes of VPI admitted in our unit before and after the availability of DM, while also taking into account feeding and growth indicators.

Since the introduction of DM, there has been a reduction in both the percentage of VPI that are fasted in their first two days of life and in the age at initiation of enteral feeding, in line with other reports in the literature (16 h in Castellano Yañez et al., 12 h in our data) [31]. The results of an international survey published in 2012 [32] showed that when DM was available, the start of feedings was earlier and faster, maybe reflecting a better gastrointestinal tolerance to human milk when compared to artificial premature formula [18,30]. Despite this, we did not see a shortening in time on parenteral nutrition or at full enteral feedings. Our findings are similar to the data reported by Corpeleijn in 2016 [33] and might explain why, unlike others [6,34], we did not see a decrease in the incidence of LOS, which is consistent with other series [22,33,35].

There has been some degree of controversy regarding the impact of availability of DM on the rates of availability of own mother’s milk, with either stability or increase being reported. In our unit, the introduction of banked milk did not change the percentage of exclusive breastfeeding at discharge, and this is in line with the observation in a slightly more mature population of preterms [31] as well as with the conclusion of a systematic review [36], which found no differences in exclusive administration of OMM on the first 28 days of life or at discharge. An Italian survey published in 2013 [13] describes that units where DM is available show higher rates of exclusive breastfeeding at discharge, although this might reflect baseline differences in the attitude towards human milk feeding. A multicentric Californian database analysis [37] observed an increase of about 10% in the rates of exclusive breastfeeding at discharge in infants under 1500 g admitted to Neonatal Intensive Care Units of mixed complexity after the introduction of DM; nevertheless, this increase was also seen in the same period of time among units that had no access to banked milk.

Milk produced by mothers that deliver preterm is richer in lipids, protein and calories [38] and is better suited for the needs of their infants regarding growth and neurodevelopment [39]. Even so, it is common practice in neonatal units to apply the same fortification protocol for OMM and DM, which is mainly supplied by mothers of term babies. This could be one of the reasons behind reported differences in growth in preterm babies fed OMM, DM and preterm formula [7,40]. A systematic review and metaanalysis [30], including 11 randomized controlled trials and 1809 patients concluded that preterms fed formula had faster rates of growth (weight, length and HC) when compared to the ones fed DM, whether exclusively or as a supplement of OMM. Interestingly, there were no differences in long term growth or neurodevelopment [30].

In our study, sequential weight, length and HC and their z-scores are similar between groups, contrary to previous reports [7,30,31,40]. What is more, the fall in weight z score at day 28 of life significantly decreased from group 1 to 2, although the difference was small (IC 95%: −1.18 ± 0.41 vs. −0.96 ± 0.66, *p* = 0.003) and disappeared by discharge time. Due to known differences in nutritional content between preterm and banked milk, this must be explained by other factors. Sisk et al. [41] also found preserved growth rates at discharge, but their classification of groups by predominant milk meant that babies in the DM group could be receiving up to 49% of their OMM. In our case, a detailed analysis exposes a slight but significant difference in early parenteral provision of nutrients, which has been shown to have an impact in growth during the first month [23].

Previous evidence points to a reduction of about 4% in the incidence and severity of NEC in VPI fed human milk [17,32,42], and this has been summarized recently [43]. It seems that the bigger the volume and the longer the duration of human milk feeding, the more impact in the occurrence of NEC [6]. Since the introduction of DM in our unit, we see a tendency to a decrease in the incidence of NEC, which is most prominent in babies born after 28 weeks of gestation, maybe because they are the ones that receive the most artificial formula (in the whole sample, 16.1% of babies born ≤ 28 weeks received some volume of formula on day 28 of life vs. 37.8% of those born at > 28 weeks, *p* = 0.002; this was 7.9% vs. 33.3%, respectively, in Group 2, *p* = 0.003). Mortality was similar before and after DM in our population as well as others [6,30], but we saw a history of NEC was more frequent in those who died in Group 1 when compared with Group 2. Surgical NEC was also less frequent in G2, although this was not statistically significant. The conclusion of a metanalysis on the impact of DM on the risk of surgical NEC was in line with this result [44]. Interestingly, if we add our numbers to the ones reported (although the metanalysis did not include observational studies), the incidence of surgical NEC would decrease from 5.2% in the group receiving formula to 1.8% in the group receiving DM, with a *p*-value of 0.002.

A recent metaanalysis [19] also describes a decrease in the incidence of BPD in DM fed versus formula fed preterm babies. Other multicentric studies [42] also found a decrease in days on mechanical ventilation or oxygen. Duration of invasive ventilation in extreme preterms (≤28 w) seemed shorter in Group 2, but the study was not powered to draw a conclusion. In any case, due to the before-after design of our study, this could merely reflect a global tendency to earlier extubation in neonatal care.

The presence of differences between sexes in the incidence of neonatal complications [45] and in growth responses to varied exposures [46] have been recently highlighted in the literature. As we mentioned, further analysis of our cohort showed that the same general conclusions apply to both sexes as well as to the whole population. When sex was introduced in the multivariate analysis, the final model did not vary and the contributing factors to NEC were gestational age, period of availability of donor milk and being SGA.

In this study, we contribute relevant information on morbidity, growth and breastfeeding outcomes of DM use in a third level neonatal unit. The single-center design should contribute to homogeneity on other aspects of care between groups. Although there is an increasing amount of literature about benefits of human milk when compared to artificial formulas, there is a high degree of variability in the methodology applied. There are differences in time windows analyzed (for example 10 days of life in Corpeleijn et al. [33]), the type of formula in the comparison group (4 standard term formula and 7 preterm formula in the 11 RCTs included in the 2018 Cochrane systematic review and metaanalysis [30]) or type of fortifier (human milk based vs. cow milk based [30]). Each center also has different criteria for the initiation, advancement and duration of DM feeding [47]. Our patients received DM under a pre-specified protocol, and we also include a detailed analysis of other growth and nutritional variables (like parenteral supply), which lacks in many other reports.

A remarkable finding of our study is that patients in the range from 28 to 32 weeks benefit more than those under 28 when using DM in terms of NEC-reduction under the conditions of our study. Most protocols of DM supplementation apply to patients born under 28 weeks GA. Even in our guidelines, patients under 28 are candidates for DM use for a longer period than those between 28 and 32 weeks. This makes us consider if this group (28–32 weeks GA) might be more sensitive to certain strategies for NEC prevention, including maybe, the use of DM, and if the results could improve with providing it for a longer time. Also, we did not find any differences in growth at discharge or in breastfeeding rates, which are two of the most reported undesired effects of using DM instead of formula for supplementation of OMM in the Neonatal Intensive Care Unit (NICU).

One of the limitations of our study is the available sample size. Also, the retrospective design does not allow for conclusions in causality. The pre- and post-period design rather than the analysis of actual intakes better reflects the impact of DM availability in a neonatal unit. Nevertheless, it should be taken into account that we have a moderately high rate of breastfeeding, so that more than half of the sample in both periods was receiving exclusively their OMM throughout admission, which might make it difficult to uncover any further differences between DM and formula. Another limitation is that the cause of preterm birth was not considered for the analysis. A higher risk of NEC has been described in some preterm subpopulations, like premature babies born after a period of intrauterine growth restriction (IUGR) [48] and they could benefit even more from the use of DM. Our study was not powered enough to detect differences in the effect of donor milk between different premature populations, which might be an area granting future attention.

## 5. Conclusions

Since the introduction of donor milk in our unit we have seen a reduction in NEC, particularly in the VPI between 28 and 32 weeks. We did not find significant differences in the incidence of other complications of prematurity or in rates of growth or breastfeeding.

Ours results support the evidence that donor milk feeding is safe and beneficial, not only for the most extreme premature babies, and that it can be implemented without impairment in nutritional outcomes while maintaining rates of breastfeeding.

## Figures and Tables

**Table 1:** Comparison of basal characteristics of the patients from groups 1 and 2.

	Whole Sample			Gestational Age ≤ 28 Weeks			Gestational Age > 28 Weeks		
	Group 1 (*n* = 99)	Group 2 (*n* = 128)	*p*	Group 1 (*n* = 26)	Group 2 (*n* = 43)	*p*	Group 1 (*n* = 73)	Group 2 (*n* = 85)	*p*
	Mean ± SD			Mean ± SD			Mean ± SD		
Gestational age (weeks)	29.5 ± 2.3	29.1 ± 2.3	0.227	26.2 ± 1.3	26.3 ± 1.3	0.853	30.7 ± 1.1	30.5 ± 1.2	0.180
Birth weight (g)	1283 ± 393	1197 ± 370	0.095	844 ± 183	854 ± 210	0.759	1439 ± 323	1371 ± 306	0.499
Birth weight z score	0.11 ± 0.87	−0.14 ± 0.93	0.204	0.05 ± 0.98	−0.12 ± 1.05	0.516	−0.00 ± 0.83	−0.16 ± 0.87	0.261
CRIB (Clinical Ric Index for Babies) score	2.2 ± 3.0	2.8 ± 3.1	0.188	5.5 ± 3.8	5.1 ± 3.3	0.622	1.0 ± 1.4	1.6 ± 2.1	0.062
	*n* (%)			*n* (%)			*n* (%)		
Gender (boy)	56 (56.6%)	69 (53.9%)	0.690	14 (53.8%)	23 (53.5%)	0.977	42 (57.5%)	46 (54.1%)	0.666
Intrauterine growth restriction (IUGR)	18 (18.2%)	15 (11.7%)	0.171	7 (26.9%)	4 (9.3%)	0.087	11 (15.2%)	11 (12.9%)	0.700
Small for gestational age (SGA)	7 (7.2%)	10 (8.6%)	0.706	3 (11.5%)	4 (10.0%)	1.000	4 (5.6%)	6 (7.9%)	0.747
Multiple pregnancy	39 (39.4%)	47 (36.7%)	0.771	8 (30.8%)	12 (27.9%)	0.800	31 (42.5%)	35 (41.2%)	0.870
Cesarean section	55 (55.6%)	86 (67.2%)	0.073	17 (65.4%)	29 (67.4%)	0.861	38 (52.1%)	57 (67.1%)	0.055
Prenatal steroid course (2 doses)	69 (69.7%)	79 (61.7%)	0.211	14 (53.8%)	29 (67.4%)	0.259	55 (75.3%)	50 (58.8%)	0.028

**Table 2:** Growth parameters of patients in Group 1 and 2.

	Whole Sample			Gestational Age ≤ 28 Weeks			Gestational Age > 28 Weeks		
Mean ± SD	Group 1 (*n* = 99)	Group 2 (*n* = 128)	*p*	Group 1 (*n* = 26)	Group 2 (*n* = 42)	*p*	Group 1 (*n* = 73)	Group 2 (*n* = 85)	*p*
Age at minimum weight (days)	3.8 ± 1.6	3.9 ± 1.5	0.612	3.9 ± 1.8	4.2 ± 1.7	0.517	3.8 ± 1.6	3.8 ± 1.4	0.982
% weight loss	11.4 ± 5.1	9.2 ± 8.6	0.026	13.8 ± 6.0	7.8 ± 5.5	<0.001	10.6 ± 4.5	10.0 ± 9.8	0.617
Age at recovery of birth weight (days)	11.1 ± 4.1	1.4 ± 5.1	0.287	11.5 ± 5.6	10.8 ± 6.9	0.663	11.0 ± 3.4	10.3 ± 3.9	0.230
Fall in weight z-score from									
birth to 28 dol	−1.18 ± 0.41	−0.96 ± 0.66	0.003	−1.33 ± 0.59	−0.91 ± 0.94	0.055	−1.13 ± 0.32	−0.98 ± 0.46	0.023
birth to 36 weeks PMA	−1.69 ± 0.79	−1.64 ± 0.70	0.652	−2.31 ± 0.95	−2.04 ± 0.76	0.217	−1.43 ± 0.53	−1.38 ± 0.51	0.588
birth to discharge	−1.42 ± 0.77	−1.42 ± 0.79	0.951	−1.96 ± 1.04	−1.86 ± 0.99	0.692	−1.23 ± 0.55	−1.21 ± 0.56	0.793

dol: Days of life, PMA: Postmenstrual age.

**Table 3:** Comparison of clinical outcomes between Group 1 and 2. Continuous variables are summarized as mean ± standard deviation and compared by Student’s *t* tests. Categorical variables are expressed as number and percentage and compared by chi-square tests or Fisher’s exact tests as appropriate.

Clinical Outcomes During Admission	Whole Sample			Gestational Age ≤ 28 Weeks			Gestational Age > 28 Weeks		
	Group 1 (*n* = 99)	Group 2 (*n* = 128)	*p*	Group 1 (*n* = 26)	Group 2 (*n* = 43)	*p*	Group 1 (*n* = 73)	Group 2 (*n* = 85)	*p*
Days on mechanical ventilation	6.4 ± 15.4	5.6 ± 10.8	0.651	21.2 ± 24.7	13.2 ± 13.8	0.141	1.1 ± 2.4	1.7 ±5.9	0.401
Days on non-invasive respiratory support	18.4 ± 21.6	21.3 ± 23.2	0.339	41.9 ± 26.3	42.3 ± 24.0	0.943	10.1 ± 11.1	10.7 ± 13.7	0.761
Days on oxygen	16.8 ± 33.0	16.3 ± 30.0	0.915	50.1 ± 49.0	42.6 ± 38.6	0.485	4.9 ± 10.4	3.0 ± 8.8	0.218
Days on antibiotics	10.8 ± 12.7	12.7 ± 12.9	0.267	24.4 ± 17.7	23.8 ± 14.4	0.884	6.0 ± 4.6	7.1 ± 7.2	0.253
Days on central line	14.4 ± 14.3	14.1 ± 10.5	0.864	25.0 ± 21.8	21.7 ± 12.2	0.420	9.4 ± 5.8	10.4 ± 5.0	0.247
Days on parenteral nutrition	13.5 ± 13.9	14.2 ± 9.7	0.658	25.8 ± 23.4	23.1 ± 12.8	0.542	10.3 ± 4.9	9.6 ± 4.7	0.308
Patent ductus arteriosus	44/99 (44.4%)	57/128 (44.5%)	0.990	21/26 (80.8%)	31/43 (72.1%)	0.418	23/73 (31.5%)	26/59 (30.6%)	0.901
Surgical patent ductus arteriosus	16/99 (16.2%)	11/128 (8.6%)	0.081	13/26 (50%)	10/43 (23.3%)	0.022	3/73 (4.1%)	1/85 (1.2%)	0.336
Necrotizing enterocolitis	9/99 (9.1%)	4/128 (3.1%)	0.055	5/26 (19.2%)	4/43 (9.3%)	0.282	4/73 (5.5%)	0/85 (0%)	0.044
Surgical necrotizing enterocolitis	5/9 (55.6%)	1/4 (25%)	0.308	1/26 (3.8%)	1/43 (2.3%)	1.000	0/73	0/85	-
Late-onset sepsis	18/99 (18.2%)	27/128 (21.1%)	0.585	14/26 (53.8%)	22/43 (51.2%)	0.829	4/73 (5.5%)	5/73 (5.9%)	0.913
Bronchopulmonary dysplasia	21/96 (21.9%)	28/124 (22.6%)	0.901	15/24 (62.5%)	25/39 (64.1%)	0.898	6/72 (8.3%)	3/85 (3.5%)	0.172
Retinopathy of prematurity (any stage)	27/87 (31%)	43/113 (38.1%)	0.302	19/23 (82.6%)	26/40 (65.0%)	0.136	8/64 (12.5%)	17/56 (23.3%)	0.103
Severe retinopathy of prematurity	5/81 (6.2%)	6/112 (5.4%)	0.809	5/23 (21.7%)	6/39 (15.4%)	0.732	0/58 (0%)	0/73 (0%)	-
Severe intraventricular hemorrhage (grade III-IV)	6/99 (6.1%)	12/128 (9.4%)	0.359	6/26 (23.1%)	9/43 (20.9%)	0.834	0/73 (0.0%)	3/85 (3.5%)	0.250
Death	4/99 (4%)	7/128 (5.5%)	0.619	3/26 (11.5%)	6/43 (14.0%)	1.000	1/73 (1.4%)	1/85 (1.2%)	1.000

## Supplementary Materials

The following are available online at https://www.mdpi.com/2072-6643/11/8/1895/s1, Table S1: Multivariate analysis by linear regression of the determinants of fall in weight z-score at 28 day of life, Table S2: Multivariate analysis by logistic regression of the determinants of NEC.
