# Supplementary material for: Availability of Donor Milk for Very Preterm Infants Decreased the Risk of Necrotizing Enterocolitis without Adversely Impacting Growth or Rates of Breastfeeding
Source: Nutrients. 2019 Aug 14;11(8):1895. doi: 10.3390/nu11081895 (PMC6722966; doi:10.3390/nu11081895)
Supplement: Supplementary file 1 [file nutrients-11-01895-s001.pdf]

Table S1: Best fit regression model for fall of z-score at 28 days of life in Group 1 and 2. After taking into account clinical and nutritional factors that influence growth (gestational age, need for oxygen during admission, IUGR, fluid intake during the first week, average protein and lipid in PN during the first week), only gestational age, IUGR, intravenous lipid during the 1st week and the Period of study were associated to fall in weight z-score at 28 dol.

Summary of model coefficients	Adjusted R square	p	Variables in the equation	Standardized (B) and 95% IC	p
Step 1	0.170	<0.001	Gestational age (weeks)<br>IUGR<br>Average lipid on PN<br>Group	0.196<br>-0.316<br>0.171<br>0.219	0.007<br><0.001<br>0.022<br>0.001

IUGR: intrauterine growth restriction, PN: parenteral nutrition, dol: days of life

Table S2: Results of the binary logistic regression analysis of the risk of NEC. We performed backwards stepwise selection, with removal based on the maximum partial likelihood estimates and starting with variables that were different between Group 1 and 2 or that were possible confounders.

Summary of model coefficients	Nagelkerke R square	p (Omnibus test)	Variables in the equation	Exp(B) and 95% IC	p
Step 1	0.294	0.002	Gestational age (weeks)<br>Group<br>SGA<br>Days on mechanical ventilation<br>Days on oxygen<br>Surgical treatment for PDA<br>Cesarean section<br>Full steroid course<br>Constant	0.639 (0.445- 0.916)<br>3.431 (0.823- 14.301)<br>11.516 (2.102- 63.103)<br>0.980 (0.920- 1.044)<br>0.994 (0.967- 1.022)<br>3.574 (0.621- 20.570)<br>1.463 (0.359- 5.967)<br>1.437 (0.367- 5.631)<br>5460.002	0.015<br>0.091<br>0.005<br>0.528<br>0.668<br>0.154<br>0.595<br>0.603<br>0.097
Step 2	0.291	0.001	Gestational age (weeks)<br>Group<br>SGA<br>Days on mechanical ventilation<br>Surgical treatment for PDA<br>Cesarean section<br>Full steroid course	0.648 (0.455- 0.924)<br>3.418 (0.824- 14.172)<br>11.427 (2.077- 62.854)<br>0.971 (0.926- 1.018)<br>3.510 (0.617- 19.961)<br>1.561 (0.392- 6.208)<br>1.438 (0.368- 5.623)	0.016<br>0.090<br>0.005<br>0.224<br>0.157<br>0.528<br>0.602

			Constant	3286.537	0.109
Step 3	0.288	0.001	Gestational age (weeks)	0.640 (0.450- 0.911)	0.013
			Group	3.749 (0.928- 15.148)	0.064
			SGA	11.545 (2.105- 63.321)	0.005
			Days on mechanical ventilation	0.972 (0.927- 1.019)	0.232
			Surgical treatment for PDA	3.358 (0.595- 18.944)	0.170
			Cesarean section	1.685 (0.441- 6.437)	0.445
			Constant	4886.0143	0.090
Step 4	0.282	<0.001	Gestational age (weeks)	0.634 (0.444- 0.904)	0.012
			Group	3.754 (0.947- 14.879)	0.060
			SGA	9.734 (1.941- 48.814)	0.006
			Days on mechanical ventilation	0.972 (0.927- 1.019)	0.232
			Surgical treatment for PDA	3.466 (0.635- 18.905)	0.151
			Constant	8208.867	0.072
Step 5	0.264	<0.001	Gestational age (weeks)	0.697 (0.509- 0.954)	0.024
			Group	3.365 (0.861- 13.148)	0.081
			SGA	7.233 (1.556- 33.628)	0.012
			Surgical treatment for PDA	1.952 (0.422- 9.020)	0.392
			Constant	554.021	0.155
Step 6	0.256	<0.001	Gestational age (weeks)	0.650 (0.496- 0.851)	0.002
			Group	4.026 (1.091- 14.847)	0.036
			SGA	8.634 (1.986- 37.535)	0.004
			Constant	4089.125	0.026

SGA: small for gestational age, PDA: patent ductus arteriosus
